# Climate Change and Human Health in Africa in Relation to Opportunities to Strengthen Mitigating Potential and Adaptive Capacity: Strategies to Inform an African “Brains Trust”

**DOI:** 10.5334/aogh.4260

**Published:** 2024-01-29

**Authors:** Caradee Y. Wright, Thandi Kapwata, Natasha Naidoo, Kwaku Polu Asante, Raphael E. Arku, Guéladio Cissé, Belay Simane, Lynn Atuyambe, Kiros Berhane

**Affiliations:** 1 Environment and Health Research Unit, South African Medical Research Council, Pretoria, South Africa; 2 Department of Geography, Geoinformatics and Meteorology, University of Pretoria, Pretoria, South Africa; 3 Department of Environmental Health, Faculty of Health Sciences, University of Johannesburg, Johannesburg, South Africa; 4 Environment and Health Research Unit, South African Medical Research Council, Durban, South Africa; 5 Kintampo Health Research Centre, Kintampo, Ghana; 6 School of Public Health & Health Sciences, University of Massachusetts Amherst, USA; 7 Swiss Tropical and Public Health Institute, Basel, Switzerland; 8 University of Basel, Basel, Switzerland; 9 Addis Ababa University, Addis Ababa, Ethiopia; 10 Makerere University, School of Public Health, Uganda; 11 Columbia University, New York, USA

**Keywords:** adaptation, development, economic resiliency, environmental health, extreme weather events, heat, mental health, public health

## Abstract

**Background:**

Africa faces diverse and complex population/human health challenges due to climate change. Understanding the health impacts of climate change in Africa in all its complexity is essential for implementing effective strategies and policies to mitigate risks and protect vulnerable populations. This study aimed to outline the major climate change-related health impacts in Africa in the context of economic resilience and to seek solutions and provide strategies to prevent or reduce adverse effects of climate change on human health and well-being in Africa.

**Methods:**

For this narrative review, a literature search was conducted in the Web of Science, Scopus, CAB Abstracts, MEDLINE and EMBASE electronic databases. We also searched the reference lists of retrieved articles for additional records as well as reports. We followed a conceptual framework to ensure all aspects of climate change and health impacts in Africa were identified.

**Results:**

The average temperatures in all six eco-regions of Africa have risen since the early twentieth century, and heat exposure, extreme events, and sea level rise are projected to disproportionately affect Africa, resulting in a larger burden of health impacts than other continents. Given that climate change already poses substantial challenges to African health and well-being, this will necessitate significant effort, financial investment, and dedication to climate change mitigation and adaptation. This review offers African leaders and decision-makers data-driven and action-oriented strategies that will ensure a more resilient healthcare system and safe, healthy populations—in ways that contribute to economic resiliency.

**Conclusions:**

The urgency of climate-health action integrated with sustainable development in Africa cannot be overstated, given the multiple economic gains from reducing current impacts and projected risks of climate change on the continent’s population health and well-being. Climate action must be integrated into Africa’s development plan to meet the Sustainable Development Goals, protect vulnerable populations from the detrimental effects of climate change, and promote economic development.

## 1. Introduction

Climate change has significant implications for human health and well-being, affecting individuals through environmental exposures and influencing the social determinants of health, such as health-related behaviors and socio-economic factors. Moreover, climate change leads to social and economic disruptions, compounding its adverse effects on human health. Environmental exposures may manifest in various ways. Direct exposures might occur during flash floods whereas indirect exposures may occur as a result of harmful changes in water, food, and air quality [1].

Several health hazards are posed by climate change. Exposure to climate change-related events such as extreme heat aggravates existing chronic conditions and increases risk of heat-related illnesses and/or mortality [2]. Wildfires, floods, and drought contribute to food insecurity, and hence undernutrition. Climate change increases the suitability of environmental conditions for infectious disease transmission [3] and can also directly impact supply of services and health systems, for example, storm damage to clinic or hospital infrastructure, or road damage preventing healthcare staff from reaching their places of work. These impacts are sometimes additive, compounding and cascading, and there are numerous modifying influences between the different pathways linking climate change and human health impacts.

Africa is deemed to be the continent worst impacted by climate change [4]. Temperatures have been rising by approximately 0.3°C/decade between 1991 and 2021, and 2021 was among the top five warmest years on record for Africa [5]. Sea-level rise along African coastlines is higher than the mean global rate and is likely to increase in the future, thereby contributing to coastal flooding in low-lying cities and increasing the salinity of groundwater [5]. Frequent droughts and extreme heat events have increased demand for water in Africa and water scarcity is likely to trigger conflict among people, especially those facing economic challenges [5].

According to the Intergovernmental Panel on Climate Change (IPCC) “climate change is already challenging the health and well-being of African communities, compounding the effects of underlying inequalities (high confidence).” [4] These impacts disproportionately affect vulnerable groups including people living in poverty, children and women, minority groups, people with pre-existing conditions, and the elderly. In sub-Saharan Africa, there are several “climate change hotspots” where strong physical and ecological effects of climate change intersect with large populations of poor and vulnerable communities. Children are especially vulnerable to the effects of climate change [6]. Children spend more time outdoors where they are exposed to heat and disease vectors such as mosquitoes and ticks; they require more water by body weight compared to adults and therefore their exposure to water-borne pathogens is higher; diarrheal disease causes dehydration in children at a much faster rate compared to adults [6].

While climate change is impacting human health and well-being in Africa, other challenges exacerbate these impacts. For example, urbanization, land use change, energy poverty, economic inequality, and external influences such as political conflict and unrest in Africa and other parts of the world affect food supply. Pandemics such as COVID-19 threaten stability and the environment due to their unprecedented multi-faceted impacts. All these factors compound the effects of climate change being experienced in African countries. Concurrently, Africa is on a development trajectory—the African Union’s Agenda 2063 calls for “The Africa We Want” through transforming economies, modernizing the agricultural section and promoting environmental sustainability, among other activities, to transform the continent into a global powerhouse of the future [7]. Grappling with climate change impacts while pushing ahead with an economic development agenda presents both challenges and opportunities for Africa and calls for increased adaptive capacity and resilience across all sectors.

This narrative review is part of the “Future of Health and Economic Resilience in Africa” (FHERA) project (https://www.hsph.harvard.edu/fhera/)—a collaboration primarily housed at the Harvard T.H. Chan School of Public Health between *The Lancet* and a core panel of experts and stakeholders from across Africa and beyond. The project is a new Lancet Commission on the Future of Health and Economic Resiliency in Africa [3]. The aim of this narrative review was to describe major climatic changes facing Africa and how they impact human health and in so doing, identify strategies to prevent or reduce adverse effects of climate change on human health in Africa. Given FHERA’s focus on economic resilience, we also considered Africa’s mitigation potential and adaptative capacity in terms of climate change-related health impacts and the continent’s current/potential resilience, especially economic resilience, is considered in the context of development and challenges facing the continent.

## 2. Methods

### 2.2 Search strategy

**Figure 1:**
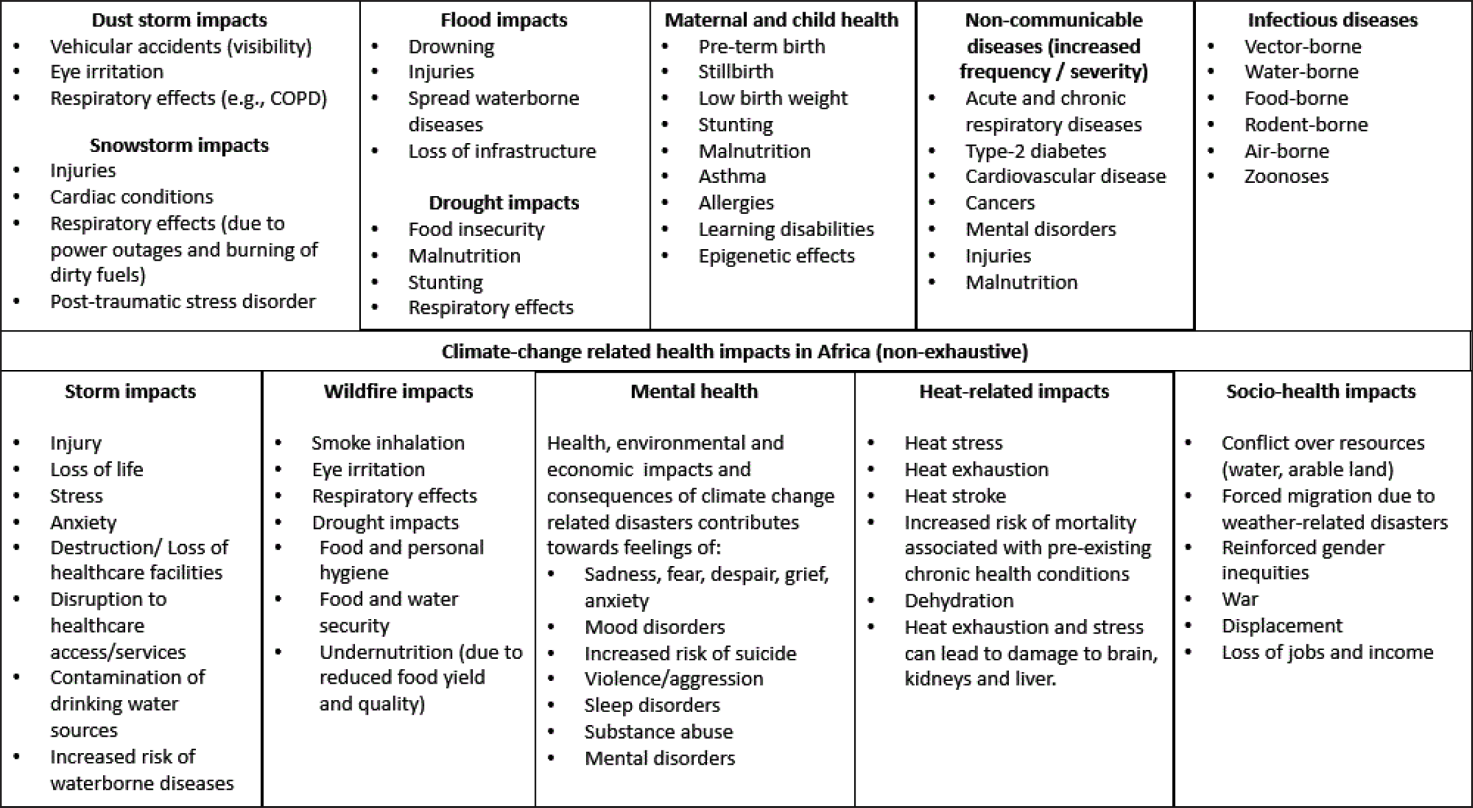
Conceptual framework applied in searching for and describing the impacts of climate change on health in Africa.

Our search for literature on climate change and human health in Africa was informed by a conceptual framework (Figure 1). The literature search was done in five relevant electronic databases: Web of Science Core Collection (accessed via Web of Science); Scopus (accessed via Elsevier); CAB Abstracts (accessed via Web of Science/OVID); MEDLINE; and EMBASE. In addition, reference lists of retrieved articles were searched manually for articles relevant to the review. The databases were searched for articles using text words for climate change and health outcomes in Africa for five years prior to 31 July 2023. The text words were *climate change, climatic impacts, drought, flood, heavy rainfall, extreme weather events*, and the like; and *health impacts, mortality, morbidity, diet, nutrition, mobility, injury, mental health*, and the like. Retrieved articles were managed in EndNote.

We supplemented our article list with materials in the grey literature. The websites of the following authorities were searched for grey literature: African Development Bank, International Development Research Centre, UK Department for International Development, United National Environment Programme, World Health Organization, World Meteorological Organization, World Bank, United Nations, United Nations Educational, Scientific and Cultural Organization (UNESCO), Intergovernmental Panel on Climate Change, United Nations Framework for the Convention on Climate Change (UNFCCC), funding bodies (e.g., Wellcome Trust), and Africa theses and dissertations, among others that we identified during this process. No content was excluded from the grey literature category, regardless of publication date and author’s country of origin (so long as the content pertained to Africa).

This was a narrative review [8] and did not strictly adhere to PRIMSA guidelines which are used for systematic or scoping reviews. Information from retrieved studies was extracted and summarized in the text of the manuscript. The goal was not to systematically gather all the evidence but instead to present relevant literature and provide a narrative around climate change health-impacts in Africa to inform the development of strategies while also reflecting on economic considerations for Africa.

## 3. Results

### 3.1 Climate change features in African regions

The African continent spans three major latitudes—the equator, Tropic of Cancer, and Tropic of Capricorn—and is the second largest continent (after Asia) at more than 30 million km^2^. There are six regions across Africa based on economic and political groupings: North Africa, West Africa, Central Africa, East Africa, Southern Africa, and the Indian Ocean Island countries (also known as Small Island States) [9]. The major drivers of climate change affecting the regions of Africa are the phases of the El Niño Southern Oscillation (ENSO) and sea surface temperature anomalies in the tropical Atlantic Ocean and Indian Ocean [5].

Climatic changes occurring in Africa vary spatially and temporally as evidenced in Table 1. All six African regions experienced an increase in average temperature from 1901–1930 to the period 1991–2021. However, the warming was slightly greater for North Africa [5]. The annual number of extreme warm days in Africa has increased since 1979 to 2021, with the number of days of mean temperatures in the warmest 1% of records ranging from one day to >14 days (the highest occurring in the year 2016) [5].

**Table 1:** General climate change risks and impacts by region in Africa. Evidence drawn from State of Climate Report (2021), State of Climate Report (2022), IPCC Africa Chapter (2021), AFDB report (2019), AFDB report (2022), and Godfrey and Tunhuma (2020).


	TEMPERATURE-RELATED IMPACTS	RAINFALL-RELATED IMPACTS	OTHER IMPACTS

**Northern Africa**	Warming the fastest, at ±0.4 °C every decade between 1991 and 2022, compared to +0.2 °C per decade between 1961 and 1990. Increases up to ±8°C in the Sahel region.	Precipitation could decline by 40–60% during summer months with risk of drought and water shortages. Shorter growing season for crops. Areas south of 25°S are projected to experience increases in precipitation.	Salinization of groundwater due to water extraction and sea-level rise, especially in deltaic areas.

**Eastern Africa**	In equatorial and tropical latitudes, mean temperatures have increased by 1–3°C over the past 50 years leading to malaria-carrying mosquitoes that survive at higher altitudes.	Increasing frequency of drought events that undermine health and livelihoods of farmers, agro-pastoralists in the region.	Environmental degradation via deforestation, intensified agriculture in arid and semi-arid lands, rapid urbanization and industrialization compound climate change impacts in the region, e.g., pest infestations due to heat affecting crops and livelihoods.

**Central Africa**	Warming has been experienced, including an increase in warm extremes and a decrease in the occurrence of cold spells. Second highest temperature rise per decade at ±0.22°C per decade compared to the pre-1960’s average.	Unlikely that rainfall will change drastically in the region, however, there is some risk of flooding for low-lying and coastal areas.	Projected median increase of 2.5 heatwave events per warm season. Larger data gaps than the rest of the continent noted in all reports.

**Western Africa**	The proportion of extremely hot days (annual maximum 35°C) and tropical nights increased by 1–9 days and 4–13 nights every decade, respectively whereas cold nights decreased.	Drought is expected to increase, and agricultural yield is projected to decline by more than 15% by 2100. Decline in rain-fed agriculture could be as high as 50%. Water stress and water scarcity are concerns.	With 15 000 km of coastline, the region is at risk of sea-level rise and coastal degradation of fisheries and tourism ventures. Malaria transmission may become unsuitable in the region due to changes in vector habitats’ conditions.

**Southern Africa**	Temperatures are projected to increase by 4–6°C by 2100. Minimum and maximum temperatures and the number of heatwaves will increase.	At 2°C warming, the region is likely to see a 20% reduction in precipitation, with more consecutive dry days and a 5–10% reduction in the volume of the Zambesi River basin.	Changing patterns in the distribution of malaria, moving from the east to the central parts of the region. Mozambique is susceptible to tropical cyclones and the north-eastern parts of South Africa to flooding associated with these cyclones.

**Small Island States**	Most African island states are experiencing rising heat and lower rainfall leading to a higher risk of drought.	Sea-level rise threatens these states together with tropical cyclones and heavy rainfall.	Loss of land to the sea means reduction in resources and space for income-generating activities.


Trends in rainfall are less consistent compared to those for temperature when looking across the six African regions. In 2021, below-normal rainfall conditions occurred over much of North Africa while north-eastern Egypt experienced above-average rainfall [5]. West Africa had a delayed onset and early cessation of rainfall while Central and East Africa as well as the Small Island States in the Indian Ocean experienced unusually high levels of rain. Other extreme and high-impact events that affect the African regions include land-falling tropical cyclones, wildfires, and sandstorms/dust storms [5].

Extreme weather and climate events are likely to increase in intensity and frequency in Africa [10] although this might not be the case for all types of events or for all places. Observed rainfall data for East Africa suggested that annual total precipitation on wet days, very heavy precipitation and severe precipitation were decreasing since 1961 [11]. Among 19 sub-Saharan African countries, excess rainfall and extreme temperatures were associated with an increase in the incidence of diarrhea and weight-for-height malnutrition among children aged 3 years and younger [12]. However, it is difficult to fully assess the impacts of these events in Africa due to the absence of an inventory of climate and weather events; developing such an inventory would assist with a better understanding of adaptation needs for the African continent.

In summary, temperature, rainfall and the occurrence of extreme climate and weather events are changing among many regions of Africa. The following sections describe the range of health impacts occurring/likely to occur as a consequence of climate change in Africa and follows the order these impacts were presented in Figure 1 (except for dust storms, and storms which are included in the section called “Impact from storms an extreme weather events”).

### 3.2 Climate change-related health impacts in Africa

#### 3.2.1 Impacts from storms and extreme weather events

Storms, wildfires, heatwaves, cyclones, floods, snowstorms, and dust storms are examples of extreme weather and climate events associated with injuries, fatalities, mental health effects, and loss and damage to infrastructure and services, including healthcare systems, among other health impacts (Figure 1) [13]. Extreme conditions in Africa, such as the drought caused by the El-Niño event in 2015–2016 in Southern Africa, and the landslide resulting from Cyclone Kenneth in Mozambique in 2019 [14] can cause physical injury, loss of life, destruction of property and population displacement. Flooding is widespread in African cities due to heavy rainfall over indigent land occupied by informal settlements or alongside rivers, and drowning of small children is common [15,16]. Since urban areas lack trees and vegetation, they are more vulnerable to extreme weather phenomena such as runoff (after heavy rainfall), dust storms (during droughts), and high temperatures [17]. In 2022, Tripoli experienced a sandstorm that caused a substantial reduction in visibility to <1 km, with severe winds ranging from 55 to 85 km/hr [18]. In Libya’s northern areas in the same year, snowstorms caused 10–20 cm of snow cover, closing major transport routes [18]. In 2021 and 2022, wildfires were linked to significant loss of life in Algeria, with 44 deaths reported in August [18]. The vast array of extreme events in Africa underscores the urgent need for comprehensive and adaptive measures to address the multifaceted health and infrastructure challenges posed by climate change.

#### 3.2.2 Impacts on maternal and child health

Maternal exposure to high temperature during early pregnancy has been associated with adverse birth outcomes, such as stillbirths, miscarriages and long-term development and behavioral anomalies [4]. Maternal exposure to heat and water scarcity are associated with an increased risk of pre-term birth, and in-utero exposure to air pollution has been associated with low birthweight and subsequent adverse consequences for the health and development of the child [19]. Heat exposure during the first trimester has also been linked with congenital anomalies and during the third trimester with pre-term birth, stillbirth and low birthweight although the biological pathways are unclear [20].

Climate change threatens the *Rights of the Child* as embodied in the 1989 Convention [21], in terms of the rights to survival, shelter, good health, well-being, education, and nutrition. Against the backdrop of efforts to prevent child morbidity and mortality in Africa, climate change has the potential to undo all the good work that has been done to protect child health and well-being. Climate threats could be direct, through extreme events such as heatwaves, floods, and droughts, or indirect, through ecosystem disruption, changes in vector patterns, air pollution, and aeroallergens [19]. The health risks for children include food insecurity and malnutrition, educational attainment, forced migration, toxicant exposure, and in-utero exposure, as well as direct morbidity and mortality from extreme weather events, infectious and respiratory diseases and diseases affecting the skin, immune, endocrinological, metabolic, and neurological systems [19].

Changes in air pollutant concentrations will likely affect child health and may result in worsening respiratory health for children suffering from asthma, allergies, and other chronic respiratory diseases. Climate change-related extreme weather events also threaten child health. In March 2019, Cyclone Idai caused destruction and healthcare service disruption in Mozambique leading to a 23% decrease in first antenatal care visits and 25% decrease in measles vaccinations that month [22].

Several determinants of maternal and child health are impacted by the effects of climate change. Food insecurity and malnutrition are leading causes of morbidity and mortality, especially in Africa. Ugandan women indicated that food insecurity was common during pregnancy and was believed to negatively impact on the health of their infant over the course of the first 1 000 days of life [23]. Representation Concentration Pathway (RCP) 8.5, often known as “business-as-usual,” is the highest baseline emissions scenario in which emissions rise throughout the twenty-first century based on modelled changes in temperature and precipitation [24]. Climate projection scenarios suggest that sub-Saharan Africa could see increased rates of stunting up to 20% although this would vary by country [25]. RCPs are trajectories of greenhouse gas concentrations used for future climate scenarios based on GHG emissions from human activities, depending on the efforts taken to limit greenhouse gas emissions. Other determinants of child health are poverty, lack of education, forced migrations and exposure to toxicants—all of these increase children’s vulnerability to the impacts of climate change. Moreover, African populations living in low-income/low-resource settings face a high burden of disease from environmental exposures and limited access to quality healthcare.

#### 3.2.3 Impacts on mental health

The association between climate change and physical health, such as flooding and infectious disease, drought and malnutrition, extreme weather events and personal injury, and so on, has been reasonably well documented; however, the exact mechanisms by which extreme climatic events such as extreme heat, floods, and droughts impact mental health remain poorly understood [26,27]. Recent efforts to understand these linkages show that affected individuals and communities experience sadness, fear, despair, helplessness, and grief which affects their mental well-being [28]. Several studies indicate an association between high ambient temperature and the exacerbation of symptoms for mental and behavioral disorders [29]. For example, higher rates of diagnosed and self-reported mental-health-related conditions, that is, substance use, anxiety and mood disorders, and psychoses, were seen during days of extreme heat [30,31,32,33]. Most suicides are related to mental illnesses [28], and some evidence links higher temperatures to increases in suicide rates [34].

According to the sixth assessment report by the IPCC, several parts of Africa are projected to experience increases in frequency and duration of droughts in future climate scenarios [4]. Droughts cause significant disruptions to agricultural production including decreased crop yields and reduced animal growth rates [35]. This leads to the loss of livelihoods and food insecurity leaving communities who are dependent on agriculture in poverty and this situation has been linked to many common mental disorders such as depression [36]. While it is widely acknowledged that Africa is one of the continents that is most vulnerable to the impacts of climate change because of its high exposure and low adaptive capacity [37], there is a lack of literary contributions from the region regarding the impacts of climate change on mental health [36]. Regardless, the mental health impacts should not be underestimated and require further research.

#### 3.2.4 Impacts on non-communicable diseases

An estimated 74% of deaths globally have been linked to non-communicable diseases (NCDs). This is equivalent to 41 million people each year worldwide [38]. LMICs experience 80% of these deaths with sub-Saharan Arica accounting for one third of these deaths [39]. East African Countries including Burundi, Rwanda, Kenya, Tanzania, South Sudan, and Uganda have also recorded significant increases in the overall NCD burden, where currently, 40% of total deaths are related to NCDs [40]. Some NCDs such as acute and chronic respiratory diseases, type-2 diabetes, cardiovascular disease, cancers, mental disorders, injuries, and malnutrition have been shown to be climate sensitive [41].

High temperatures due to climate change-induced warming decreases the availability of clean and accessible water sources [42], while rising sea levels, unpredictable rainfall and extreme climate events lead to low agricultural output [43]. These climate change-related environmental determinants of health influence food insecurity, economic stability, and poverty which are three risk factors that exacerbate NCDs [44]. Therefore, the projected increases of climate hazards across Africa are likely to worsen an already existing health crisis further by increasing the disease profile of NCDs.

#### 3.2.5 Climate change-related heat-health impacts

About a quarter of the 5 million annual deaths globally associated with non-optimal temperature occur in Africa [45]. Africa is expected to experience disproportionately more health impacts due to heat exposure than other continents with projections of heat-related mortality likely to increase in the middle-east and northern parts of Africa, in particular among people > 65 years (the elderly) when considering 2°C global warming [46].

Heat stress is also likely to be higher in certain African settings, such as in schools without energy-intensive cooling systems like air-conditioning, outdoor occupational settings and prisons, known to be overcrowded and poorly ventilated [47]. Dwellings made from poorly insulated and inadequately ventilated materials are also risky; these are found in urban slums and informal settlements which exist in majority of African cities and pose a direct risk to their inhabitants’ health in the context of global warming [4]. Moreover, the lack of green space and the heat island effect make urban areas especially vulnerable to heat-health impacts. Emerging evidence is also pointing to the impact of heat stress on productivity loss as evidenced from a study on workers from the floriculture industry in Ethiopia [48].

#### 3.2.6 Impacts on infectious diseases

Climate change, especially global warming, and changes in precipitation, influences the dynamics (epidemiology, geography, risk of) of vector-borne, water-borne, food-borne, rodent-borne, and airborne infectious diseases [49]. Each of these diseases is discussed below.

##### Vector-borne infectious diseases

Warmer temperatures and changes in rainfall patterns will continue to influence the incidence and distribution of malaria in Africa. Currently, about one third of the continent is optimally suitable for malaria transmission all year round [4]. Projections for malaria transmission under RCP 4.5 and RCP 8.5 climate change scenarios suggest there will be an emergence of new endemic malaria vector hotspots, increased prevalence and spatial extent increases. RCP 4.5 is described as a moderate scenario in which emissions peak around 2040 and then decline [24]. Temperature increases projected for Africa under both RCP 4.5 and RCP 8.5 show that countries expected to be the worst impacted by increased risk of malaria transmission in 2030 include Northern Angola, southern DRC, western Tanzania and central Uganda [4]. This is because warmer conditions influence malaria transmission by increasing the development and survival rate of malaria parasites and their vectors. In other parts of the continent, changing climatic conditions and decreased environmental suitability will no longer be suitable for the malaria vector and parasite, for example, in Burkina Faso, Cameroon, Ivory Coast, Ghana, Niger, Nigeria, Sierra Leone, Zambia and Zimbabwe. Rising temperatures in these countries will mostly exceed optimum temperatures reaching thresholds beyond which the adult population of mosquitos will decrease significantly (under RCP8.5) [4].

Dengue, Zika, and Rift Valley Fever have been observed at higher altitudes compared to earlier times because of temperatures warming, as well as occurring during dry periods when open water storage and stagnant water present as breeding sites, and during flooding which spreads viruses through human settlements [50]. Epidemics of dengue, chikungunya and yellow fever are projected to increase in the future with shifts in mosquito species. However, if temperatures become too high, this may prohibit transmission of some infectious diseases. Yellow fever is estimated to cause 78 000 deaths each year in Africa and is projected with high confidence that annual deaths in Africa will increase in 2050 [51].

##### Water- and food-borne infectious diseases

Water- and food-borne diseases include salmonellosis, cryptosporidiosis, norovirus, cholera, among others, that often result in diarrhea symptoms in affected individuals. Diarrheal diseases are among the leading causes of death in children under 5 years of age in Africa [52]. Changes in temperature and precipitation may increase transmission of bacterial and protozoal diarrheal disease agents via contamination of water and food.

Cholera incidence has been shown to increase with increasing temperature. In the summer of 2021, a cholera outbreak occurred in Niger and Nigeria because of flooding compounded by inadequate waste management, poor sanitation practices, lack of drainage systems and human consumption of contaminated water [5]. In particular, the outbreak affected children aged 5 to 14 years and more than 3 000 people died, this despite efforts to curb cholera in these countries and there being no observed cholera cases since 2018 [5]. Access to adequate, clean water and the means to practice health-hygiene, such as those promoted via Water, Sanitation and Hygiene (WASH) are important to curb the spread of infectious diseases especially in light of possible disruptions to water availability and sanitation from infrastructure failure/breakdown due to extreme weather events [4,53]. Impacts on other infectious diseases such as schistosomiasis, and salmonella will also affect human health in Africa.

##### Rodent-borne infectious diseases

The available information on zoonotic pathogens in Africa is limited, particularly concerning the role of rodent reservoirs in transmitting diseases [54]. A wide range of zoonotic pathogens, including bacteria, viruses, and protozoa act as receptor hosts and key vectors in rodent reservoirs [55]. In European, Asian, and American contexts, rodents are known to transmit various diseases such as leptospirosis, Sin Nombre virus, plague (*Yersinia pestis*), and Hantaan virus [56]. These diseases typically spread through dust contaminated with dried rodent urine [56,57,58] and fecal material [55]. In Africa, diseases such as Lassa fever [59], leptospirosis [60], hantavirus [61], and plague [62] are transmitted by rodent reservoirs. However, there is a lack of focused studies analyzing the impact of climate change on rodent reservoirs and the spread of these diseases in the African context.

Lassa fever is endemic to rural West Africa, and other countries in Africa [63] with rodent reservoirs common around fields and villages [59]. An estimated 100 000–300 000 people contract Lassa fever in endemic areas annually [63]. Reported cases have been steadily increasing over the past two decades, and factors such as increasing rainfall and agricultural expansion may expand the suitable habitat for rodent reservoirs [59]. Future shifts in rainfall patterns could affect reservoir host population cycles and the seasonality of human risk [59]. Integrating ecological forecast models into health planning could enhance preparedness for potential surges in Lassa fever in West Africa [59]. Although Lassa fever is caused by rodent-borne virus, the virus can be transmitted from human-to-human through contact with body fluids and aerosol droplets [63].

Leptospirosis poses a global threat, with the highest human exposure occurring in indigent tropical communities in the tropics [59]. Reports from the WHO Leptospirosis Epidemiology Reference Group (LERG) indicate that leptospirosis incidence may be high in Africa, but also highlight the lack of available data [60,64,65]. Flooding after extreme weather events can lead to large human outbreaks, and climate change is exacerbating the frequency and intensity of such events [59]. Agricultural expansion and unplanned urbanization increase rodent-human contact and susceptibility to flooding. Although about ten rodent species are known as leptospirosis reservoirs in Africa [60], the effects of climate change on these niche reservoirs remain unknown.

Hantavirus, introduced to Africa in the last fifteen years, has not been widely studied on the continent [61]. Entry through identified seaports suggests that brown rat-associated hantavirus can readily propagate in Africa [61]. Further studies are needed to analyze the effects of climate change on rodent reservoirs and the potential spread of hantavirus.

Plague outbreaks result from various interacting factors, including population density, the life cycle of vectors and rodent reservoirs, and environmental conditions. Climate factors such as temperature and rainfall can influence the behavior and survival of fleas and rodent reservoirs [66], including the survival and reproduction of fleas [67,68,69]. In cold areas, low winter temperatures can harm rodent populations [70]. and the survival of the plague-causing bacteria in rodents that hibernate or lie dormant during certain seasons can also influence the occurrence of the disease [71]. Local climate factors, influenced by larger phenomena like El Niño and the Indian Ocean Dipole, affect the spread of plague in Madagascar [72]. Higher temperatures and increased rainfall are associated with a rise in plague cases [62]. Studies in LMICs show that the seasonality of specific transmission profiles confirms the optimal environmental conditions required for the flea and rodent species [62]. These seasonal patterns are yet to be characterized in Africa to clarify the impacts of climate change on the occurrence of plague in the future.

##### Airborne infectious diseases

Airborne diseases are caused by transmission of droplets of pathogenic microbial agents expelled through coughing, sneezing, or through close personal contact [73]. Most airborne infectious diseases are viral agents such as strains of influenza and coronavirus. Bacterial agents that cause respiratory diseases such as *Mycobacterium tuberculosis* and *Bordetella pertussis* are a significant public health concern. *Cryptococcus meningitis, Pneumocystis jirovecii*, and other species of fungi have recently been highlighted as public health threats [74]. In addition to temperature and extreme events, other factors influenced by climate change that play crucial roles in transmission of airborne pathogen transmission include exposure to ultraviolet light, humidity, and wind [75].

Meteorological factors affect the dispersion of fungal spores. The gradual rise in temperatures, attributed to climate change, enhances the resilience of fungal spores to high temperatures, and extreme weather events contribute to the spread of pathogenic fungal spores [74,76]. The peak levels of fungal spores during spring and autumn align with optimal temperature and humidity conditions [77]. An examination of skin test frequencies in Cape Town, South Africa, disclosed *Alternaria* as the primary fungal allergen among children with respiratory symptoms, with *Aspergillus, Cladosporium*, and *Epicoccum* also identified as significant sensitizing fungal spores [77,78].

A study on recurrent admissions to a pediatric intensive care unit for acute severe asthma revealed a higher incidence of sensitization to *Aspergillus* and *Cladosporium* in the ‘seasonal’ group compared to the control group [77,79]. Instances of flooding in Botswana, Mozambique, Zimbabwe, and South Africa posed a public health threat due to the presence of mold spores in buildings [77]. Countermeasures such as drying walls, cleaning with household bleach, and employing facemask respirators with filters for cleaning and rescue teams were implemented [77,80].

The expanding population of immunocompromised individuals, particularly those with human immunodeficiency virus, is increasingly susceptible to opportunistic fungal pathogens like *C. meningitis* and *P. jirovecii*, resulting in significant respiratory morbidity [74]. New fungal species identified in Africa raise concerns about their prevalence and potential to cause disease in immunocompromised patients [74]. The spectrum of infection symptoms varies from mild and undetected to more severe conditions such as pneumonia, acute respiratory distress syndrome, often culminating in disseminated disease [74]. Importantly, the long-term consequences of climate change on the future spread of diseases caused by fungal pathogens remain uncertain.

Changes in climate, encompassing temperature, humidity, and rainfall fluctuations, influence how tuberculosis patients react by altering vitamin D distribution, exposure to UV radiation, malnutrition, and other risk factors [81]. The surge in severe weather events causes people to relocate, increasing the number of at-risk populations prone to tuberculosis thereby creating conditions favoring tuberculosis transmission and development and interrupting diagnostic and treatment efforts [81]. Thus, climate change affects tuberculosis transmission, especially in LMICs where tuberculosis is already a significant public health concern [81].

A global study investigating meteorological factors associated with rotavirus transmission reveals varied transmission routes, including airborne dispersal, and viral survival on soil and surfaces [82]. The impact of changing weather patterns on rotavirus disease burden differs based on climate zones [82]. While detailed observations and surveillance offer insights into transmission routes, they have limited predictive potential [82]. Targeted studies in diverse settings are crucial for anticipating climate change’s impact on rotavirus spread [82].

Significant differences in seasonal patterns of Acute Respiratory Infections (ARI) were seen in two neighboring refugee camps in Kenya [83]. Viral transmission observed in Dadaab displayed distinct seasonality, peaking in November and December [83]. In Kakuma, no seasonality was observed despite higher hospitalizations for Severe Acute Respiratory Infections (SARI) [83]. These variations may be linked to geographical locations [83]. Kakuma reflects equatorial region trends and Dadaab mirrors climatic trends in the Horn of Africa [83]. Similar seasonal differences between neighboring tropical countries in Africa and Asia have been previously reported [83]. Surveillance points are needed to comprehensively assess temporal and climatic variations in ARI caused by viral illnesses in Africa [83].

#### 3.2.7 Vulnerability factors influencing climate change health risks

There is substantial evidence emphasizing the link between climate change and conflict. For example, populations living in conflict-affected areas are more vulnerable to the impacts of climate-change related events, because they have fewer resources to respond, mitigate or recover from climate shocks [84,85]. Health and social services, economic opportunities and food systems are often lacking or unreliable in these regions [86]. Adverse climatic conditions also exacerbate the impacts of unstable economies, conflict, and pandemics on food security with projections for Africa showing that more than 400 million Africans are anticipated to be undernourished by 2030 [4]. The ND-GAIN Country Index which measures climate vulnerability and adaptation readiness based on several indicators reported that 60% of the twenty countries considered to be most vulnerable to climate change are sites of armed conflict [84]. According to the ND-GAIN Country Vulnerability rankings, Somalia, a country that experiences many climatic shocks (prolonged droughts, rising temperatures, cyclones, sand and dust storms) and has endured decades of ongoing armed conflict, is scored as the most climate vulnerable country out of 185 countries [87]. This is a stark reminder of the increased exposure and vulnerability of people living at the intersection of climate change and conflict. However, while conflict exacerbates the effects of climate change, climate change also indirectly drives conflict. It is often described as a “risk multiplier” because it worsens factors that are known to increase conflict risk [88]. These include social and economic exclusion, a history of conflict and grievances, environmental degradation, and competition over access and management of depleting natural resources [89].

Internal and cross-border migration and displacement are another rising concern of climate change impacts with floods, storms and drought contributing the most to disaster-related displacement [90]. This humanitarian crisis is unfolding across the globe. In Africa, Somalia, Ethiopia and Kenya continue to experience the longest and most severe drought on record and more than two million people have been displaced as a result. In 2018, severe tropical cyclones Idai and Kenneth occurred in Malawi, Mozambique and Zimbabwe a mere six weeks apart causing death, injuries, disease and destruction [91,92]. An estimated 2.2. million people were displaced in the aftermath. In the first half of 2023 alone, over 100 000 people fled the Bay region of southern Somalia due to the region’s worst drought in 70 years, while more than 260 000 people in the Hiraan region were displaced by flash flooding [93].

Another social risk factor of health that is amplified by climate change is gender inequality. Women are more vulnerable to the effects of climate change for several reasons. For instance, agriculture is the one of the most important employment sectors for women in Africa, however reduced agricultural activity and poor yields due to drought and floods restricts women’s income and the ability to provide for themselves and their households [94]. Also, women are displaced due to climate change-related disasters and are more vulnerable to human trafficking, sexual exploitation and other forms of violence [95].

In summary, the impacts of climate change on human health in Africa are evident and span a wide range of health outcomes. Vulnerable groups include children, women, people living in areas of conflict, among others. While some impacts are well researched, such as heat-health impacts, others such as rodent-borne diseases and NCDs require further research to fully comprehend the interplay between climate change and the likely adverse impacts on human health in Africa. For some health impacts, potential interventions have been identified; however, the effectiveness of these adaptation measures (for example, ensuring access to water for good hygiene to curb the spread of waterborne diseases) has not been well documented in the literature. The following sections consider the economic implications of climate change-related health impacts and then focus on solutions and actions to protect health and well-being in Africa.

## 4. Economic implications of climate change-related human health impacts in Africa

Climate change is causing an increase in adverse health impacts which are costly to treat thus place a strain on African countries’ already limited healthcare budgets. Climate change may also lead to an increase in healthcare costs and widen the divide in existing inequities in health and healthcare delivery. Low-income people, indigenous communities, people living in fragile environments, and people who are experiencing homelessness are particularly at risk [96].

As temperatures rise, outdoor workers are less productive, and this can negatively affect the economy [48]. For instance, farmers may not be able to work as long or as efficiently in extreme heat, which can reduce crop yields and affect food security. Other workers, such as healthcare professionals and first responders may have difficulties gaining access to their places of work during extreme weather events thus jeopardizing access to care among communities. Climate change-related health problems such as heat-related illnesses and disease outbreaks can discourage tourism to Africa which can have a significant impact on the economies of countries that rely on tourism such as South Africa, Tunisia, Tanzania, Kenya, Morocco, and Egypt [97].

Climate change is also causing changes in weather patterns that can have a devastating impact on the livelihoods of farmers and fishermen. For example, droughts can cause crops to fail, and rising sea levels can destroy fishing communities due to saltwater intrusion into freshwater reserves [98]. This can lead to the loss of livelihoods which can have long-term economic impacts on communities and the country. Increased government spending on disaster relief is highly likely in a changing climate. Climate change is increasing the frequency and severity of natural disasters such as floods and storms which can require significant government spending on relief efforts. This can divert resources away from other economic development projects and activities.

## 5. Air pollution-related health impacts and co-beneficial solutions for climate change

Ambient air pollution, which is increasing in Africa, was estimated as being responsible for almost 400 000 deaths in Africa in 2019 [99]. Air pollution is a major contributor to cardiovascular [100,101] and respiratory diseases [102,103,104], which can lead to premature death.

In a changing climate, air pollutants and pollutant concentrations are likely to change. For example, warm temperatures and sunny skies can increase ground-level ozone concentrations. In turn, personal exposure to ozone can trigger adverse health impacts such as chest pain, coughing and throat irritation, and worsen bronchitis, emphysema, and asthma [105]. Other pollutants that may increase in concentration in a changing climate are particulate matter which is associated with health risks at every stage of the life course [106]. This has grave implications for the formation of human capital and productive workforces. The loss of economic output in 2019 due to air pollution related morbidity and mortality was estimated at $3.2 billion in Ethiopia, $1.63 billion in Ghana and $349 million in Rwanda [99]. It is evident that deliberate interventions are required to ensure economic resilience in Africa in the face of climate change and air pollution threats.

A report entitled “Integrated Assessment of Air Pollution and Climate Change for Sustainable Development in Africa” [107] projects that Africa could prevent 880 000 deaths every year by 2063 by acting on air pollution and climate change and addressing five key areas: transport, residential, energy, agriculture, and waste. Recommended actions would lead to reduced carbon dioxide, methane, and nitrous oxide emissions, and improve food security by reducing desertification and increasing rice, maize, soy, and wheat yields.

In the absence of deliberate interventions, air pollution and climate change will increase morbidity and mortality, impair human capital formation, and undercut development [99]. Addressing air pollution in Africa will have multiple co-benefits including improved public health. In Africa, a “baseline scenario” of high material consumption, population growth and warming were compared to projected scenarios (low warming and high warming) [108]. It was projected that under a low warming scenario (for climate change) annual premature deaths due to fine particulate matter (PM_2.5_) are reduced by ~ 515 000 by 2050 relative to the high warming scenario (100 000, 175 000, 55 000, 140 000 and 45 000 in Northern, West, Central, East and Southern Africa, respectively) [108].

Another co-benefit from reducing air pollution is increased agricultural productivity which can increase food security and improve likelihoods. Many of the sources of air pollution, such as burning of fossil fuels and biomass, also contribute to climate change. Therefore, addressing the air pollution problem can help mitigate the effects of climate change. Transitioning away from “dirty fuels” to clean and renewable energy sources helps to reduce air pollution and improves energy security, reducing dependence on fossil fuels. Air pollution has negative effects on economic productivity by reducing worker productivity and increasing healthcare costs. By significantly reducing air pollution, the national economy will benefit.

## 6. Vulnerability, adaptive capacity, resilience, and suggested strategies to protect health in Africa

In this section, we unpack the aspects affecting vulnerability, adaptive capacity, and resilience of Africa in relation to climate change and health and draw together three overarching strategies with suggested actions (Table 2), drawn from the literature reviewed in section 3 and the discussion here, to help guide decisionmakers when tackling climate change and health in Africa.

**Table 2:** Strategies for addressing climate change and health challenges in Africa.


STRATEGY	TIMING	DESCRIPTION	SUGGESTED ACTIONS

Integrated approaches to health in Africa should be pursued to deliver multiple health benefits for humans and ecosystems	***Medium- to long-term***	The intersections between climate change, air pollution and human health involve interactions of numerous systems and sectors. Such complexities require holistic and cross-sectoral approaches like One Health and Planetary Health to ensure long-term effectiveness of responses to health risks.	Inter-departmental/inter-ministerial collaboration in policy, strategy and planning within and across countries for systemic change to bolster adaptive capacity in the health sector and healthcare systems. Mindset shift in roles, responsibilities, and prescribed areas of jurisdiction to adopt all-encompassing action plans rather than working piecemeal. This also requires a change in management methods from working in isolation to participatory and increased community involvement Consideration of emerging risks and uncertainties; not necessarily as a separate plan but integrated into planning to support prioritization and garner support (including funding support)—flexible and adaptive national, regional, and continental planning structures/thinking to be responsive and receptive to new developments and changes. Building individual and institutional capacities for initiating and sustaining the implementation of the selected integrated approaches Concerted effort to destigmatize mental illnesses in Africa. Strategies to support those suffering from or most susceptible to mental illness is crucial to avoid a health crisis in the face of climate change. Policy changes that reduce environmental impacts of climate change combined with promotion of healthy lifestyles to effectively reduce disease burden.

Africa should build substantially upon progress made in recent decades towards the Sustainable Development Goals by investing in solutions that collectively reduce the impacts of climate change and air pollution on health	***Short- to medium-term***	Critical areas for attention include poverty, inequality, restoration and protection of ecosystems and forests, increasing food security and agricultural productivity, adopting clean energy, and improving waste management.	True implementation of the African Union’s Agenda 2063 with accompanying financing, capacity strengthening and political support across the continent. Development/environment intersection—green (renewable solutions especially for alleviating energy poverty) solutions. Identification and implementation of Co-benefits and shared solutions (climate change and air pollution). Collaboration of the health sector with other sectors to increase health co-benefits. This requires the availability of skilled human-capacity and institutional structures within health departments able to communicate, understand the interconnections of the other sectors with health and propose the relevant solutions.

Special attention is needed to address water stress and the impacts of extreme weather and climatic events in Africa	***Short- to medium-term***	Water stress, droughts and floods undermine water security and aggravate conflict and displacement, and the consecutive disasters affect the mental health of an ever-increasing segment of the population. Not only does this threaten lives and livelihoods but such impacts affect stresses negatively impact economies and ecosystems. Preparedness and enhancing adaptive capacity are needed to bolster Africa’s efforts to prevent adverse human health and well-being impacts.	Raising awareness and literacy about climate change and air pollution towards transformative climate response, especially in places impacted by one or the other, or both, hence the intersection of experience and perception of change then affects risk perception and urgency. In the presence of climate services that provide information to inform a response, people are more likely to act, adapt and cope with climate change and air pollution impacts. Community awareness campaigns/climate change/air pollution knowledge drives to improve uptake of suggested solutions. Surveillance and climate services such as early warning systems for heat, floods, drought. Public advisories via meteorological services/weather services. Strengthening the capacity of communities and the health sector in anticipating and managing mental health problems in vulnerable/exposed/affected communities. Climate extreme events compounding with so many other risks affect not only injuries and mostly known communicable diseases, but also the mental health of more people.

Research, surveillance and upgraded data capture and processing is critical to provide the evidence required to inform data-driven decision-making and policy development	***Short- and medium-term***	Drivers of climate change and its health impacts are complex, multi-sectoral and prohibitively expensive as they operate under both short and long time periods. Given the need for context specific primary evidence and the complex multi-dimensionality of data needed to fully examine the related issues, significant investments are critically essential to build human capacity, enhance physical infrastructure (to collect, analyze, and interpret data) in order to conduct strategically designed studies, put together effective policies to protect human health, and enforce policy mandated actions in ways that are optimized for economic resiliency.	Training multi-disciplinary climate scientists in all components of investigation—including health, data science, and implementation science. Investing in monitoring, computing, and enforcement infrastructure. Funding support dedicated to cohort studies in Africa to be able to collect study population-specific disease burden, health effects (i.e., NCDs, air pollution and heat impacts) for a localized context to inform targeted climate change adaptation actions/strategies. More climate and mental health population studies in Africa are needed to develop suitable interventions.


Africa is particularly vulnerable to the impacts of climate change due to its agro-based economies, poor infrastructure, and low levels of technology and industrialization. Other factors include geography and political stability. Subsequently, Africa has low adaptive capacity (defined as “the ability of a system(s)/ region to evolve in order to accommodate climate changes or expand the range of variability with which it can cope”) (IPCC, AR4 WGII Chapter 6) to cope with the adverse direct and indirect effects of climate change. Climate change further exacerbates the vulnerability of the African population due to mass migration and displacement of people who already lack the resources to adapt to their environments [109].

In addition, the political tensions associated with scarcity of food and water compound their social vulnerability and need of international protection. Climate-induced migration could also amplify pre-existing stresses related to poverty, informality, social and economic exclusion, and poor governance—all of which have significant impacts on the vulnerability and adaptive capacity of the continent [110].

Nevertheless, there are several successful capacity strengthening initiatives underway, some examples of which are given here. Small-scale farmers in Cameroon practice agroforestry (planting of trees/shrubs on farms) and monoculture as their main adaptive choices [111]. Their capacity to adapt was enhanced with access to credit, household income, number of farms, access to information and access to land. Among coastal women in Zanzibar (Tanzania) adaptive capacity, namely assets, flexibility, organizations, learning and agency, was low with extended poverty and very high dependence on seaweed farming that was being severely affected by climate change [112]. They needed to diversify their livelihoods, and additional education and training to increase knowledge was essential.

Preparedness and awareness of the impacts of climate change are key components of adaptive capacity to prevent adverse health impacts from climate change. In southern Africa, several examples illustrate countries’ adaptation activities, from early warning alert and response systems for health surveillance to capacity strengthening programmes for national decision-makers. Other examples of successful adaptation strategies are the use of drought-resistant crops, improved irrigation systems and the development of climate-smart technologies. Strengthening Africa’s adaptive capacity will also depend on its access to resources and technology, political will and leadership, and the ability of communities to work together to implement effective, locally-appropriate adaptaton strategies [113].

The effectiveness of adaptation strategies is linked to resilience. This highlights the important role of climate resilient development, a process of implementing mitigation and adaptation measures to support sustainable development [114]. The *IPCC Assessment Report Six Chapter 9: Africa* recommends five key dimensions for climate resilient development in Africa: climate finance, governance, cross-sectoral and transboundary solutions, adaptation law (to support climate finance flows and prevent maladaptation, for example) and climate services and literacy. All dimensions require attention.

More than 250 million Africans and almost half of Africa’s countries are deemed to be in transition, striving to move from fragility (resulting from conflict, war, corruption etc.) towards resilience [115]. Resilient African populations and economies have key characteristics including light and power, food, industrialization, integration (transport networks) and improved quality of life for all people. Strengthening resilience in Africa should be underpinned by three core elements: capacity building and technical assistance, arrears clearance, and supplementary financing. These would help to deepen knowledge and understanding of tailored responses and mitigation of fragility. Many African countries are in the relatively early stages of development presenting them with the opportunity to pursue non-polluting pathways to growth (avoiding fossil fuel-based economies). With far-sighted investment in renewable energy and clean technology, countries can avoid generating ambient air pollution and contributing to climate change [116].

Efforts to enhance Africa’s power and water infrastructure are essential to help build climate resilience. Africa has a large untapped hydropower potential and is estimated to be currently only using around 10% of its technical potential [117]. Harnessing renewable energy options will support access to essential resources and services in times of climate shocks. Infrastructure also needs to be designed and developed considering life span and potential climate change threats, such as river basin flooding and storm surges that threaten water carriage between urban and rural areas. In so doing, Africa will achieve its twin goals of economic development and climate resilience.

Furthermore, Africa needs community-based primary health and hospital systems development that is integrated with strengthened public health systems for prevention and response to disease outbreaks and climate emergencies. Africa also needs to foster domestic resource mobilization and savings, and boost information and communication technology, among other activities, to accelerate implementation of the Sustainable Development Goals (SDGs) and the African Union’s Agenda 2063. Almost 300 million children in Africa do not have school infrastructure for school attendance [118]. Loss of biodiversity/forestry and land degradation is ongoing and requires strong public-private partnerships to halt such loss. Partnerships need earnest strengthening with ongoing lags in domestic revenue generation and foreign direct investment. Africa faces poor debt management and has lost billions of dollars to illicit financial flows; both problems require urgent attention for Africa’s economic resiliency to be bolstered. There are, however, success stories of projects/activities aimed at achieving the SDGs that have positive spin-offs to protect human health impacts associated with climate change. Many of these were executed among low-income communities where interventions including social protection (e.g., public works programs, cash transfers, social insurance and microinsurance schemes), universal healthcare, and climate-smart buildings and agriculture go a long way to improve quality of life and create resilience among communities.

## 8. Conclusions

We set out to review the literature on climate change and health impacts in Africa, as well as the implications of such for economic development considering Africa’s current and potential adaptive capacity. Drawing together the evidence and suggesting strategies and actions, we provide a ‘brains trust’ for African leaders and decision-makers to consider as they strive to meet the SDGs, secure ‘The Africa We Want’ and protect the African populations from the harmful impacts of climate change. Significant effort, resources and commitment are needed to achieve these goals but with pressing climatic impacts already jeopardizing African health and well-being and projected to continue to do so in the future, it is now time for climate action to be integrated into Africa’s development trajectory.
